# Employment for Children

**Published:** 1871-07

**Authors:** 


### EMPLOYMENT FOR CHILDREN.

BY UNCLE BOB.

Every parent should see to it that some employment is arranged for the children of the family.

Whenever a child attains to such an age that it can understandingly perform some of the many little household duties of its parents, it is old enough to have some of them assigned to it to do.

Suppose the child is a boy, six or seven years of age. He is old enough to undertake something more than his plays or games. He can assist his mother in many and various little ways. A time should be assigned him for doing these duties, for the point to be accomplished is, to awaken a habit of uniform attention to labor. Spasmodic spurts of work have little value.

The parent needs to see that attention is given to the manner in which these duties are fulfilled. Look after the child, and by the interest which is shown, cause him to see that the charge is not to be neglected, or done in any manner he sees fit.

Perhaps, if you are the parent, you may have a feeling that you would much rather do the work yourself. No doubt you would do it far quicker and easier. It is for the sake of the child that you have given it to him. He is to be made to feel that he is of use, and that he must take part in his own care and support.—As he increases in years he will easily take upon himself additional duties, until his labor is found to be a considerable part of the daily share for all.

Of what use is it for a young man of fifteen, sixteen or even eighteen years of age, to tell how much books he knows and not be able to tell with this the share he takes in the family cares.

Let him learn to do errands, with accuracy and politeness, between the families of the neighborhood. Let him before school, instead of playing the entire time, perform, if necessary, an hour's work of chores about house.

I know that the majority of readers of this article will be families where there are many members.

As I would advise for the employment of one, so I would advise for the employment of every child in it capable of understanding duty. Apportion all these little helpers their share; see to it that they are instructed in the best manner of doing that part, and that it is well done.—By your ready help and encouragement show that you are not a taskmaster, but a friend.

If you who read this and are struck with the possible truth of what I say, will you not give it a really earnest thought? Stop and consider whether you are doing by your family all that you ought. Are your children occupied in play or study only? Do they have a regular work to do, if for not more than half an hour? If they do not, you may be certain that you are not doing right by them.

Neatness, self-confidence, politeness and frankness, all spring from a habit of regularity and faithfulness. An honest, working, industrious boy of fourteen, has more real manliness and therefore trustworthiness, than an unsettled, unoccupied one of eighteen or even twenty.

Right here is a very important thing which I want to add. Give your boy this regular routine of usefulness, and you can tell nine times in ten, at any time of day, where to find him.—You *are in duty bound* to know *just where your son is*, and what he is doing at any half hour of the day. No parent is excusable in the least for a neglect to know this. Children love to slip off and be their own masters. Human nature leads to this feeling of self-reliance, and it is easiest met by finding occupation at once useful and entertaining. Vice comes with idleness to every boy. I care not how busy you may be as a parent, you are *never* excusable if you can't "place" your boy whenever asked.

Much of what has been said is applicable to the girls in the family. By nature and rules of society they are more domestic, and more likely to be in the way of rendering the mother assistance. Still, there are many girls who are not brought up to systematic attention to labor.—They need the same watchful care that is necessary for the boys of the family.

Now that I have said so much for the parents and grown up people, I feel disposed to give the children themselves some advice. In the next Bistoury I will talk directly to the little folks about a number of things which I want them to know and remember.

---

FOOLS VS. IMPS OF MISCHIEF.—A certain old teacher used to remark that he would rather have "ten devils in a class than one fool." He could make something of the imps of mischief, not by suppressing their jollity, but by turning it into right channels. The "fool" is not troublesome, but hopeless; he lacks energy of mind. A friend of ours, an experienced teacher, says: "The hardest working, most brilliant and successful student I ever had the pleasure of teaching, was a young man whom the president of the college called a monkey, too full of frolic to accomplish any thing useful. He *was* too frolicsome to do any thing soberly, more especially if gravity was insisted on as a duty. But when his overflowing humor was allowed to brighten his work, he was the most persistent student in the institution; he made fun of labor that sober-sided plodders broke their hearts and deranged their stomachs over."—*College Courant.*

---

JEWISH PHYSICIANS IN ROME.—Although Roman History mentions a large number of celebrated Hebrew physicians who attended former Popes in cases of severe sickness, and although Leo X.'s body physician was a Jew, the practice of medicine is at the present time allowed to Jews only on the condition that they confine themselves to members of their own religion. A Hebrew doctor, who two years ago attended to a Catholic who had fainted in the street, and visited him at his special request, at his home, escaped punishment only through the intercession of some influential persons. The practice of pharmacy in Rome is absolutely prohibited to Jews.—*Pharm. Zeitung.*

